# Hyperinsulinemic Hypoglycemia in Three Generations of a Family with Glucokinase Activating Mutation, c.295T>C (p.Trp99Arg)

**DOI:** 10.3390/genes12101566

**Published:** 2021-10-01

**Authors:** Aleksandra Gilis-Januszewska, Anna Bogusławska, Artur Kowalik, Ewelina Rzepka, Karolina Soczówka, Elwira Przybylik-Mazurek, Bogusław Głowa, Alicja Hubalewska-Dydejczyk

**Affiliations:** 1 Department of Endocrinology, Endocrine Oncology and Nuclear Medicine, Medical College, Jagiellonian University, 31-008 Cracow, Poland; boguslawskaania@gmail.com (A.B.); ewelinaj.rzepka@gmail.com (E.R.); karolina.wachacka@gmail.com (K.S.); eprzybyl@cm-uj.krakow.pl (E.P.-M.); berkerus@gmail.com (B.G.); alahub@cm-uj.krakow.pl (A.H.-D.); 2 Department of Molecular Diagnostics, Holycross Cancer Center, 25-734 Kielce, Poland; Artur.Kowalik@onkol.kielce.pl; 3 Division of Medical Biology, Institute of Biology, Jan Kochanowski University, 25-406 Kielce, Poland

**Keywords:** Familial Hyperinsulinemic Hypoglycemia (FHH), *GCK* mutation

## Abstract

Familial Hyperinsulinemic Hypoglycemia (FHH) is a very rare disease with heterogeneous clinical manifestations. There are only a few reports of heterozygous activating mutations of glucokinase (*GCK*) attributable to FHH, with no reports describing effects in the course in pregnancy with affected mother/affected child. A large kindred with FHH and *GCK*:c.295T>C (p.Trp99Arg) pathogenic variant was identified in which four family members from three generations were affected. The clinical follow up in one clinical center lasted up to 30 years, with different times of diagnosis ranging from neonate period to adulthood. The severity of hypoglycemia was mild/severe and fasting was the trigger for hypoglycemia. Response to diazoxide varied from good, in the neonate, to moderate/poor, in childhood/adulthood; however, this was biased by poor compliance. Treatment with somatostatin analogues was discontinued due to side effects. Over time, patients developed clinical adaptation to very low glucose levels. During pregnancy, episodes of severe hypoglycemia in the first trimester were observed, which responded very well to steroids. The clinical course of the *GCK*:c.295T>C (p.Trp99Arg) mutation varied in the same family, with the development of clinical adaptation to very low glucose levels over time. Treatment with steroids might prevent hypoglycemia during pregnancy in an affected mother.

## 1. Introduction

Familial Hyperinsulinemic Hypoglycemia (FHH) is a very rare disease characterized by excessive insulin secretion from pancreatic β-cells independent of glucose serum level. The heterogeneous clinical manifestation of the disease causes a risk of late diagnosis or even misdiagnosis. In infants and children, it can lead to serious and permanent damage to the central nervous system. Familial Hyperinsulinemic Hypoglycemia has been correlated with pathogenic variants in 15 genes: *ABCC8*, *KCNJ11*, *GCK*, *GLUD-1*, *HADH1*, *SLC16A1*, *HNF4A*, *HNF1A*, *UCP2*, *PMM2*, *CACNA1D*, *HK1*, *PGM1*, *FOXA2* and *EIF2S3*. These mutations have been associated with approximately 48% of cases [1]. The genetic background of the remaining cases is unknown. Other systemic diseases such as Kabuki, Rubinstein-Taybi, Sotos, Beckwith-Wiedemann, Costello and Turner syndrome can present with hyperinsulinemic hypoglycemia as a major symptom [2]. Depending on which locus bears a pathogenic mutation, the disease-associated loci differ in phenotype and pattern of inheritance. Heterozygous activating mutations of Glucokinase *(GCK)* have been reported to be the rarest cause of hypoglycemia attributable to hyperinsulinism. To date, 22 different *GCK* pathogenic variants have been reported, and these reports involved a limited number of families. Most of these cases were identified because of family histories with hypoglycemia having dominant patterns of transmission [3,4,5,6,7,8,9,10,11,12]. The course of hyperinsulinism related to the *GCK* mutation might vary from a mild form of hypoglycemia, which can be managed with diazoxide as reported in most cases, to a severe clinical phenotype of uncontrollable hypoglycemia leading to death [13]. Glucokinase catalyzes the first step in glucose metabolism in pancreatic β-cells and the liver and serves as the β-cell glucose sensor [14]. Heterozygous mutations that reduce enzyme activity can cause a subtype of maturity-onset diabetes of the young 2 (MODY2) [15], while heterozygous activating mutations cause hypoglycemia [16]. Expression of these.activating mutations shows increased affinity for glucose with elevations in calculated enzyme activity indexes and decreased calculated glucose thresholds for insulin release [17,18]. We present the long-term clinical characteristics of a family with the activating mutation *GCK*:c.295T>C (p.Trp99Arg) rs1554335751, including a description of the course of the disease during pregnancy in an affected mother/affected child. In addition, comparison with previous reports of patients with *GCK* activating mutations [3], including three patients with a mutation leading to the same amino acid exchange as the family presented here, gives us a greater understanding of this very rare disease. 

## 2. Materials and Methods

**Figure 1:**
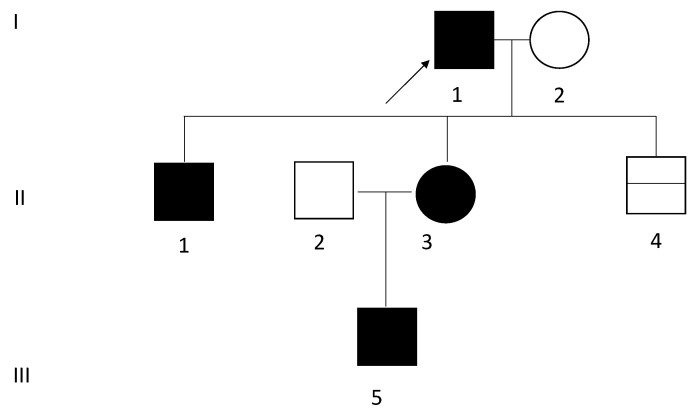
Pedigree of a family with Familial Hyperinsulinemic Hypoglycemia; (FHH) with Glucokinase (*GCK*) mutation. Generations available for the study are indicated by Roman numerals I–III. Black symbols indicate an affected subject. Squares represent male; circles represent female. The arrow indicates the index patient. The horizontal line in square II.4 indicates a negative *GCK* variant gene test.

A kindred was identified in which four family members from three generations had clinical symptoms of hyperinsulinemic hypoglycemia (a pedigree of this family is presented in Figure 1).

Genetic sequencing in affected family members and clinical, biochemical, and metabolic assessments were performed. Our study is part of the project “Genetic determinants of hyperinsulinemic hypoglycemia”. This study followed Good Clinical Practice guidelines and the guidelines of the Declaration of Helsinki. The study protocol was approved by the Jagiellonian University Ethics Committee. The committee’s reference number is KBET/175/B/2014. All study participants gave their written informed consent prior to participation in the study. In cases of children under the age of 18, a parent gave their written informed consent prior to participation in the study.

Biochemical assessments using different biochemical methods were performed during hospitalization or during outpatient clinic visits. Potential secondary causes of hypoglycemia (adrenal and somatotroph pituitary axis insufficiency) were excluded. Repeated abdominal ultrasonography, computed tomography (CT), magnetic resonance imaging (MRI), and somatostatin receptor imaging (SRI) with 99mTc were performed in patients I.1, II.1, and II.3. Scintigraphy using a 99mTc-labelled glucagon-like peptide-1-analogue (99mTc-GLP-1) was performed in patients I.1 and II.3 prior to genetic screening to determine the focal/ diffuse type of FHH and the possible need for surgery. Clinical course of the disease was presented based on medical records and current medical assessments, including 4-h oral glucose tolerance tests (OGTT) for the index patient I.1 and patient II.1. Symptoms of hypoglycemia were described as mild or severe according to current diabetes case guidelines [19].

### 2.1. Genetic 

Molecular genetic studies were carried out using DNA isolated from peripheral blood leukocytes from five family members. Isolation of the DNA was performed using a QIAamp DNA Mini Kit (Qiagen, Germantown, MD, USA,) and treated by RNase (AppliChem, Darmstadt, Germany). 

The genetic panel included 12 genes. Eight genes were directly associated with FHH: *ABCC8*, *KCNJ11*, *HADH1*, *GCK*, *GLUD1*, *SLC16A1*, *UCP2*, *HNF4A*. Three genes were relevant for the most frequently occurring diseases with phenotypes similar to FHH: *G6PC, SLC37A4, INSR*. To exclude the possibility of insulinoma as a component of multiple endocrine neoplasia type 1 syndrome, the *MEN1* gene was tested. The custom panel, including the above-mentioned genes, was designed using the Ion Ampliseq designer (LifeTechnologies, ThermoFisher Scientific, Waltham, MA, USA). The gene panel was sequenced using high-throughput sequencing.

After isolation, DNA from each sample was used for library preparation. Libraries were obtained with the use of Ion AmpliSeq™ Library Kit 2.0, Custom Panel, and Ion Xpress Barcode Adapters 1-16 Kit (LifeTechnologies, ThermoFisher Scientific, Waltham, MA, USA.), according to the manufacturer’s recommendations (Ion AmpliSeq Library Kit user guide—LifeTechnologies, ThermoFisher Scientific, Waltham, MA, USA).

Sequencing was carried out on an Ion S5 sequencer (LifeTechnologies, ThermoFisher Scientific, Waltham, MA, USA).

### 2.2. NGS Data Analysis

Raw NGS data were processed with Torrent Server Suite 5.2 (LifeTechnologies, ThermoFisher Scientific, Waltham, MA, USA). The obtained sequences were mapped against the human reference genome (hg19). Identification of variants was performed using the Variant Caller v5.2 program, with standard parameters for CHPv2: minimum allele frequency—SNP = 0.02, INDEL = 0.05, minimum quality = 10, minimum coverage = 20. Variant Caller was connected to the Integrative Genomics Viewer (Broad Institute, Oakland, CA, USA).

The presence of a potentially pathogenic variant was confirmed by Sanger sequencing in each family member. The exon of interest was amplified using HotStarTaq DNA Polymerase (Qiagen, Germantown, MD, USA) and then was sequenced by conventional Sanger sequencing using the BigDye^®^ Terminator v3.1 Cycle Sequencing Kit (LifeTechnologies, ThermoFisher Scientific, Waltham, MA, USA.). Samples were then analyzed on a sequencer (Genetic Analyzer 3500 Series (Hitachi, Tokyo, Japan), LifeTechnologies (ThermoFisher Scientific, Waltham, MA, USA)).

### 2.3. Sanger Sequencing Data Analysis

Sequences were aligned using SeqScape v2.7 software (LifeTechnologies, ThermoFisher Scientific, Waltham, MA, USA) against the reference sequence (LRG_1074) from the NCBI GeneBank database. The identified variants were analyzed according to recommendations from the American College of Medical Genetics [20] and compared against the following databases: NCBI dbSNP, NCBI ClinVar, and the Human Gene Mutation Database (HGMD).

## 3. Results

A large Polish kindred was identified in which four family members from three generations had a heterozygous variant in *GCK* c.295T>C confirmed by genetic testing. According to ACMG recommendations, with the help of Varsome (varsome.org.), variant c.295T>C was classified as pathogenic [20]. This variant was absent in population databases, including Exome Aggregation Consortium (EXAC) and gnomAD. An alternative variant in the same codon c.297G>C (Trp99Cys) was classified as pathogenic by UniProt Variants (and confirmed using ACMG) and predicted to be deleterious by MutationTaster, MVP and PolyPhen. The above-mentioned variant was also present in the ClinVar Database (with 2 submissions, 5 publications: PMID: 12941786, PMID: 15677479 PMID: 17082186, PMID: 18370929 and PMID: 18450771) and was classified as a pathogenic variant. The index patient I.1 (Figure 1) had undergone genetic testing at the age of 54 years.

Table 1 shows clinical outcomes in the presented family with mutation (*GCK*:c.295T>C (p.Trp99Arg). Clinical observation and follow up in our Department was conducted for over thirty years. 

### 3.1. Patient I.1 

**Figure 2:**
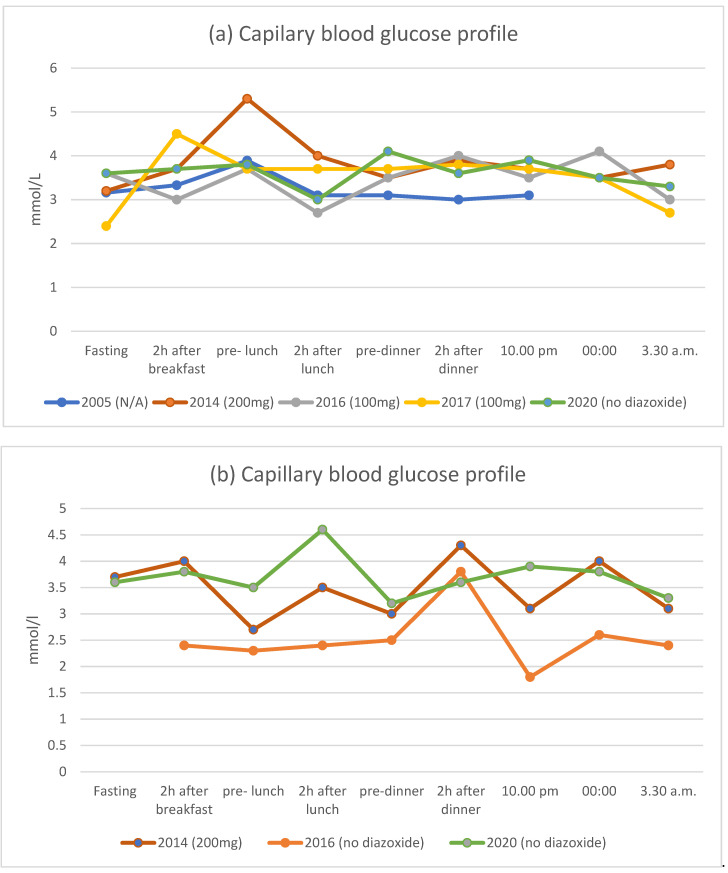
Capillary blood glucose profiles (mmol/L) of index patient I.1 (**a**) and his son II.1 (**b**).

**Figure 3:**
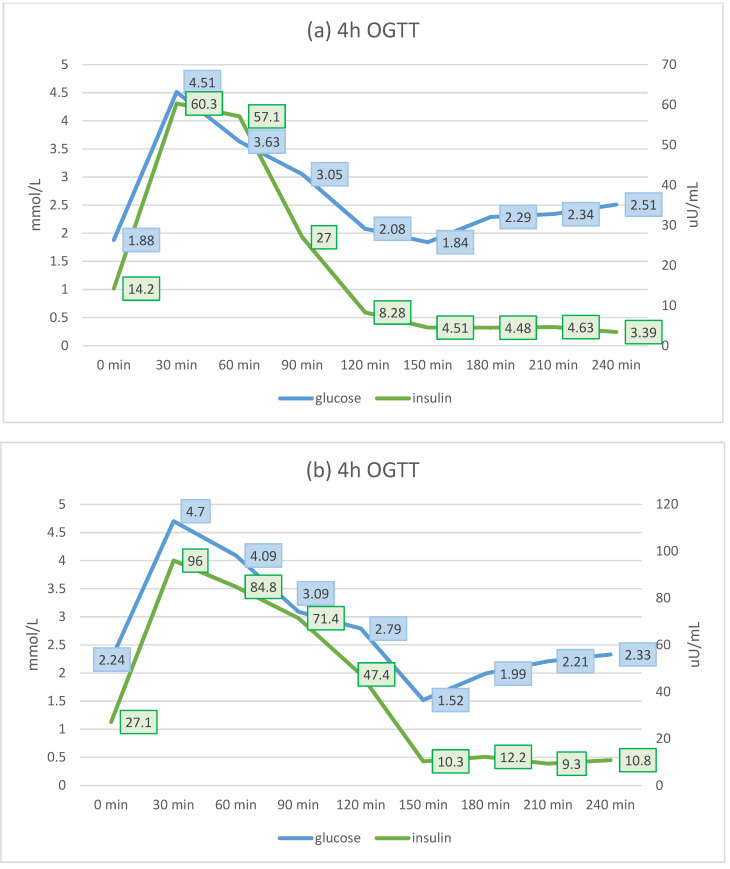
Blood glucose (blue) and insulin (green) levels during four-hour OGTT in index patient I.1 (**a**) and his son II.1 (**b**). OGTT: oral glucose tolerance test.

Patient I.1 is a 57-year-old male, (birth weight 3800 g, current BMI 32 kg/m^2^), father of three children (two affected), who was diagnosed with hyperinsulinemic hypoglycemia at the age of 20. According to his medical history, symptoms of hypoglycemia were present from the postnatal period, with a significant increase in intensity during early childhood. The patient’s parents, aged 88 and 84 years, had no history of hypoglycemia and did not undergo genetic testing. During childhood, the patient had learning and behavioral problems. No correlation between hypoglycemia and physical activity was observed. Hypoglycemic episodes occurred both during fasting and after meals, and their severity ranged from mild to serious. The patient experienced many severe episodes of hypoglycemia with loss of consciousness and injuries. Epilepsy was diagnosed at the age of 10 years, with the probable trigger of these seizures being episodes of hypoglycemia. As of publication, the patient was being treated with carbamazepine (400 mg per day). Diazoxide treatment was initiated at the age of 20 years, which decreased both the number and severity of hypoglycemic episodes; however, poor patient compliance was noted (Table 1 and Table 2). There were no potential adverse events associated with diazoxide reported. Optimal glycemic control was observed while receiving regular treatment with diazoxide 200 mg (Table 2, Figure 2a). Additionally, the course of the disease appeared to be affected by the patient’s BMI. A greater BMI was associated with better glycemic control, HbA1c level, and resolution of neuroglycopenic symptoms, regardless of the patient’s adherence to treatment (Table 2, Figure 2a). His most recent 4-h OGTT, performed at the age of 57 years, showed typical fasting hypoglycemia with hyperinsulinemia, followed by a decrease in glucose level with an excessively high insulin level 2–3 h after oral glucose load (Figure 3). Subsequently, a very slow, spontaneous increase in glucose level associated with a stable level of insulin was observed. The lowest glycemic values were observed during fasting (1.88 mmol/L) and at 150 min (1.82 mmol/L). During the OGTT, no clinical signs of hypoglycemia were observed in the patient. Over time, the patient developed clinical adaptation to very low glucose levels and did not present with any clinical signs of hypoglycemia. We did not observe any threshold glucose level which led to alarming signs or symptoms in this patient.

**Figure 4:**
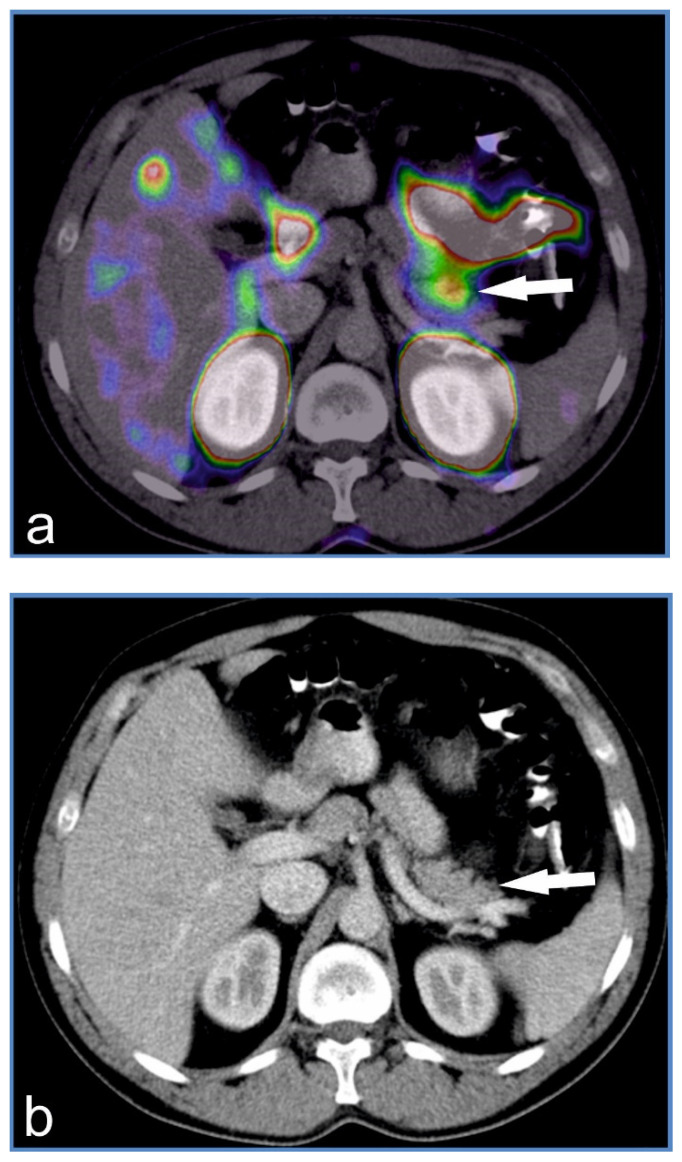
Diffused uptake of tracer found in the body/tail of the pancreas observed via 99mTc-GLP-1 scintigraphy (**a**) and transverse abdominal computed tomography imaging of the pancreas (**b**) in patient I.1 [21].

The patient declined to undergo pancreatectomy, which was offered in early adulthood. Imaging studies including MRI and SRI with 99mTc were unremarkable, while in GLP-1 scintigraphy, diffuse tracer uptake was observed in the head and tail of the pancreas (Figure 4). Lipid profile and liver enzymes were within normal range (Table 3), while his HbA1c was 4.1%. Abdominal ultrasound revealed an enlarged liver (163mm) without steatosis.

### 3.2. Patient II.1

A son of affected patient I.1, aged 29 years (birth weight 4400 g, current BMI 25.9 kg/m^2^), Patient II.1 was diagnosed at the age of four. However, according to his medical history, symptoms of hypoglycemia were present from the postnatal period, with significantly increased intensity in early childhood. The patient had difficulties at school and experienced problems with concentration. Hypoglycemic episodes were present both during fasting (with overnight hypoglycemia) and 2–3 h after meals. Furthermore, hypoglycemic episodes were not correlated with physical activity. 

Diazoxide treatment was initiated at the age of 4 years with partial improvement in the number and severity of episodes; however, poor compliance was noted as the patient took the medication irregularly. There were no potential adverse events of diazoxide reported. The patient’s parents refused consent for pancreatectomy, which was offered in childhood. Optimal results in the capillary blood glucose profile were observed in 2020, despite poor compliance, as the patient was not taking diazoxide during this time (Table 1 and Table 4; Figure 2b). The course of the disease appeared to be affected by the patient’s BMI; a greater BMI was associated with better results in the capillary blood glucose profile and HbA1c levels, regardless of the patient’s adherence to the treatment. His most recent OGTT, performed at the age of 29 years, showed typical fasting hypoglycemia with hyperinsulinemia, followed by a decrease in glucose level with an excessively high insulin level 2–3 h after oral glucose load (Figure 3b). Subsequently, a very slow spontaneous increase in glucose level together with a stable insulin level was observed. Fasting glycemia was 2.34 mmol/L, while the lowest glucose concentration was observed at 150 min (1.5 mmol/L). During the OGTT, the patient did not present with any clinical signs of hypoglycemia up to 120 min. After this time, he became distracted. 

Over time, the patient developed clinical adaptation to very low glucose levels, presenting with milder symptoms of hypoglycemia. As of publication, his glucose setpoint for triggering signs of hypoglycemia such as irritation, distraction, and blurred vision was 2 mmol/L. The patient reported that his symptoms were milder in comparison to early adulthood. He participated in several dietary consultations and reported that avoiding fasting, having frequent meals with short intervals between meals, and having a late-night meal prevented further episodes of hypoglycemia. His lipid profile and liver enzymes were within normal range (Table 3) and HbA1c level was 3.8%. Magnetic resonance imaging of the pancreas was unremarkable. 

### 3.3. Patient II.3 

A daughter of affected patient I.1, aged 26 years (birth weight 3650 g, current weight 68 kg, BMI 24.2 kg/m^2^, previous BMI at the age of 23 was 22.3 kg/m^2^), Patient II.3 was diagnosed at the age of three. Her reported glycemia was 1.28 mmol/L and diazoxide treatment was initiated. Symptoms of hypoglycemia were more pronounced in early childhood and the severity of her hypoglycemic episodes was mild to severe. There was no correlation between hypoglycemia and physical activity. Hypoglycemic episodes were present both during fasting and 2–3 h after meals. Diazoxide treatment was continued; however, poor compliance was noted, as the patient took the medication irregularly. There were no potential adverse events of diazoxide reported. In 2015, she stopped received treatment with diazoxide. At the age of 19 years, treatment with somatostatin analogues was attempted. However, due to the presence of gastrointestinal side effects and a lack of consent for the continuation of treatment, the medication was discontinued. The patient’s parents refused consent for pancreatectomy, which was offered during childhood. The patient had difficulties at school and experienced problems with concentration. Additionally, epilepsy was diagnosed at the age of nine (seizures were seen during normoglycemia) and treatment with sodium valproate was initiated. Magnetic resonance imaging of the pancreas was unremarkable, while in GLP-1 scintigraphy diffuse uptake of the tracer was seen in the head and tail of the pancreas. 

The patient became pregnant in 2015. She was not being treated with diazoxide at the time of conception or during pregnancy. During early pregnancy, many hypoglycemic episodes (mild to severe) occurred with reported glucose levels down to 1.39 mmol/L. Steroid therapy (Hydrocortisone 10 mg 3 times daily) was initiated, with good response. The minimal glucose level rose to 2.4 mmol/L. In the second and third trimesters of pregnancy, there were no severe episodes of hypoglycemia. After delivery, no treatment was received, and the patient experienced many hypoglycemic episodes with reported levels down to 1.67 mmol/L. Spontaneous resolution of her symptoms was observed three months postpartum. Since 2016, the patient has had no medical follow-ups, has not been treated with diazoxide, and experiences mild hypoglycemic episodes with no important clinical manifestations. As of publication, her usual fasting glycemia was 3 mmol/L. During the day, glycemic levels varied between 3.3 to 3.6 mmol/L. Over time, the patient developed clinical adaptation to very low glucose levels, presenting with milder symptoms of hypoglycemia. Her current glucose setpoint for triggering symptoms of hypoglycemia such as distraction, headache, or blurred vision was 2.7 mmol/L. The patient reported that her symptoms became milder in comparison to early adulthood. Dietary interventions used for the prevention of hypoglycemia included avoiding fasting, having frequent meals with short intervals between meals, and having a late-night meal. 

### 3.4. Patient III.5 

Patient III.5, the son of II.3 (grandson of I.1) was born in 2015 (birth weight 3850 g, glucose level after delivery was 0.83 mmol/L). Familial hyperinsulinism, based on family history, was diagnosed in the postnatal period with immediate initiation of diazoxide treatment. Patient compliance was good. Diazoxide therapy at a dose of 8–9 mg/kg/day was successful in maintaining normoglycemia. There were no potential adverse events of diazoxide reported. As of publication, the patient used a continuous glucose monitoring system. 

Since the age of four, the child has been receiving medical support due to developmental and psychological difficulties. 

Poor compliance was seen in affected family members I.1, II.1, and II.3 in terms of regular medical follow-ups and regular treatment with diazoxide.

## 4. Discussion

In our study, we present a long-term observation of a three-generation family with FHH and *GCK*:c.295T>C (p.Trp99Arg). To the best of our knowledge, this is the first time that this disease has been described in the course of pregnancy in an affected mother and child. Our data suggest a variable disease course over time with the development of adaptation to hypoglycemia. 

Familial Hyperinsulinemic Hypoglycemia is a very rare disease and its prevalence varies across different populations. In Europe, FHH occurs at a rate of 1:50,000 [22]. In most patients, signs of hypoglycemia are observed shortly after birth. However, in some cases, the first manifestation of the disease might be diagnosed in late infancy or early childhood [1,17]. In patients with FHH, hypoglycemia might be observed not only during fasting but also after a meal or exercise [11]. Additionally, the severity of hypoglycemic symptoms might vary between patients and even between members of the same family. The severity of these symptoms could also become milder over time [11,17,23]. In some cases, FHH can be diagnosed late due to misdiagnosis; for example, repeated vegetative symptoms of hypoglycemia with fainting might be misdiagnosed as epilepsy and treated with anticonvulsants. As a consequence, irreversible damage to the central nervous system could lead to intellectual disabilities and epilepsy. 

The heterozygous activating mutation of glucokinase is among the rarest reported cause of hyperinsulinemic hypoglycemia. In a study of non-syndromic children with hypoglycemia, the prevalence of activating *GCK* mutations was estimated to be 1.2% (2 out of 167 cases) of all FHH patients [16]. In patients with *ABCC8-* and *KCNJ11*-negative FHH that display good response to medical treatment, the prevalence of *GCK* mutations rises to 7%. At present, there are 22 different *GCK* reported pathogenic variants (Table 5).

Most of the reported *GCK* mutations were missense variants (12/21); however, an insertion mutation was also noted (1/21). Similar to the family presented here, most *GCK* cases were identified because of a positive family history of hypoglycemia with a dominant pattern of transmission. Early diagnosis in patient III.5 just after birth, related to known family history, allowed for immediate initiation of treatment with diazoxide, which will, it is hoped, prevent any significant impairment of the nervous system. In most reported cases of the *GCK* mutation leading to hyperinsulinism, varied severities of hypoglycemia have been reported, ranging from mild to severe clinical phenotypes of uncontrollable hypoglycemia [13].

Christesen et al. [16] described two children who were diagnosed with the equivalent variant in the same codon as in the presented family, *GCK*:c.295T>A (p.Trp99Arg). The first male patient was a full-term birth, with a mass of 3080 g, and was diagnosed one day after delivery. This patient had hypoglycemia that was difficult to treat and required the maximum dose of diazoxide (up to 20 mg/kg/day) plus additional treatment. His father was a mutation carrier with a milder phenotype (lowest glucose level 2.7 mmol/L [3,16], diagnosed at the age of 33 years via cascade genetic screening). The second male patient was born at full-term, with a body mass of 4000 g, and was diagnosed with the *de novo GCK*:c.295T>A (p.Trp99Arg) mutation one day after delivery, presenting with a milder phenotype (lowest glucose level 2.1 mmol/L, requiring lower doses of diazoxide without the need for additional medication). Treatment with diazoxide 8–9 mg/kg/day successfully controlled the hypoglycemia. Both patients had a continued need for medication throughout childhood and were categorized as diazoxide-responsive. The patients were followed up until they reached the age of 18 years [3,16]. 

In our presented family, the severity of hypoglycemia appeared to differ among the affected family members, with the index patient having the most severe course. The common pattern seen in adult family members was early presentation (confirmed in the neonatal period in patient III.5), severe hypoglycemia in childhood, and some improvement in early adulthood. Additionally, a common symptom was hypoglycemia triggered by fasting. However, a complete assessment of the course of hypoglycemia was difficult due to different times of diagnosis, different times of diazoxide initiation, and poor compliance. Poor compliance and/or an incomplete response to diazoxide could explain hypoglycemic complications such as learning and behavioral problems during childhood. Late diagnosis, lack of treatment, and poor compliance could explain the advanced course of the disease in the index patient, who experienced many episodes of severe hypoglycemia along with related traumas and epilepsy. In reported cases of the *GCK* mutation, phenotypes of patients differed depending on whether it was a *de novo* mutation or a familial case. A more severe disease course was observed among patients with *de novo* mutations having a poor response to diazoxide. Additionally, more than half of the *de novo* cases were characterized by a higher incidence of macrosomia. These were mostly diagnosed during the neonatal period (5/7 cases), while another two patients were diagnosed at the ages of nine and 17 years. The age at onset of symptoms in the autosomal dominant group varied from the time of birth to 44 years; however, episodes of hypoglycemia were noted earlier in most patients. Similar to other published cases, our patients were diagnosed at various ages, from neonate to adulthood. The highly variable age of diagnosis is likely to be influenced by several factors including the functional severity of the mutations, the genetic background, and delays in diagnosis. 

A similar report, describing a mild disease course among three family members with a newly diagnosed mutation *GCK*:c.269A>G (p.Lys90Arg), was recently published by Fan Ping et al. [18]. The index patient was a woman diagnosed at the age of 20 years with asymptomatic fasting hypoglycemia (2.9 mmol/L). Her birth weight was 4.6 kg, current BMI was 20.4 kg/m^2^, and physical and mental health were reported as excellent. Cascade genetic screening of the family revealed that her mother and aunt were also affected. Both were found to have asymptomatic fasting glycemia [18]. 

Over time, patients I.1, II.1, and II.3 became clinically adapted to very low glucose levels with mild to no symptoms. Moreover, they were not aware of their hypoglycemia. We can speculate on the mechanisms related to this phenomenon. Analyzing historical data (Table 1, Table 2, Table 3 and Table 4) and current blood glucose levels, it appears that the course of the disease in adult patients is affected by BMI, with greater BMIs being associated with better results in capillary blood glucose profiles and HbA1c levels, regardless of the patient’s adherence to the treatment. This suggests a role for the possible influence of insulin resistance. This mechanism could also be confirmed by the clinical improvement noted at the time of transition (early adulthood) [27]. Another mechanism which might explain the adaptation to very low glucose levels was observed in patients with type 1 or type 2 diabetes mellitus. Recurrent hypoglycemia has been shown to reduce the patient’s awareness of hypoglycemia and to reduce the threshold glucose level which precipitates the counter-regulatory response necessary to restore euglycemia during subsequent episodes of hypoglycemia [28]. 

In patient II.5, at the age of 18, somatostatin analogues, known as another possible treatment option for hypoglycemia, were introduced to supplement diazoxide treatment. However, they were not continued mainly due to gastroenterological complications. In all patients, except for III.5, severe hypoglycemia, partial response to diazoxide, and poor compliance were seen. Because of this, pancreatectomy was offered; however, none consented to the operation.

There have only been a few cases of pregnant patients with FHH reported in the literature, and none have involved an affected mother/affected child with *GCK* mutation, which suggests that the diagnosis and treatment of this disease are rather challenging. Our patient, who was not receiving treatment with diazoxide at the time of conception, experienced many episodes of mild to severe hypoglycemia in the first trimester. Since the patient refused diazoxide treatment during pregnancy, steroids were initiated with a very good response observed in the second and third trimesters. Interestingly, after delivery, despite the fact that no treatment was being received, the patient experienced many episodes of hypoglycemia which spontaneously resolved after three months. Good response to steroids during pregnancy and hypoglycemic episodes after delivery might also suggest the importance of insulin resistance in the course of the disease. There are several reports regarding octreotide therapy during pregnancy; however, these have conflicting results. Octreotide administration during pregnancy in hyperinsulinism may pose a risk of fetal growth retardation [29,30].

A strength of our study is that we analyzed the long-term clinical follow-up of a substantial number of family members having the same extremely rare mutation. Furthermore, to the best of our knowledge, this is the first description of the course of the disease during pregnancy in a *GCK*-affected mother/affected child. We reviewed all reported *GCK* mutation cases, including presentations of all familial cases.

A weakness of our study is poor patient compliance, which can create a bias in the assessment of diazoxide effectiveness and its influence on the disease course.

In summary, we identified a three-generation family with FHH and Glucokinase-Activating Mutation c.295T>C (p.Trp99Arg) and presented a long-term follow-up study including a description of the course of the disease in pregnancy in an affected mother/affected child. Furthermore, a review of reported cases, including familial cases, showed that *GCK*-FHH has a varied clinical course with variable response to medical treatment, and this was similarly noted in the presented family. 

## 5. Conclusions

Early diagnosis and immediate initiation of adequate treatment in neonates as well as during pregnancy in *GCK*-mutation FHH-affected patients might decrease the frequency and severity of hypoglycemic episodes. Long-term follow-up of *GCK*: c.295T>C (p.Trp99Arg) FHH patients suggests the development of clinical adaptation to very low glucose levels over time, with no hypoglycemic awareness and involving mild to no symptoms. Treatment with steroids might be an effective approach in the prevention of hypoglycemia during pregnancy in an affected mother. 

## Figures and Tables

**Table 1:** Clinical characteristics of affected family members.

Patient	I.1	II.1	II.3	III.5
Sex (male/female)	male	male	female	male
Birth weight (g)	3800	4400	3650	3850
Age at the time of diagnosis (yr)	20	4	3	postnatal period
Severity of hypoglycemia	from mild to serious	from mild to serious		
			from mild to serious	mild
Pretreatment plasma glucose	NA	2 mmol/L	1.28 mmol/L	0.87 mmol/L
Response to diazoxide	good to poor *	poor *	poor *	good

* assessment was biased due to poor compliance. NA: data not available

**Table 2:** Summary of metabolic profile results, concomitant symptoms of hypoglycemia and treatment in index patient IA.

Year	Age	BMI	Weight (kg)	Fasting Blood Glucose (mmol/L)	Insulin (uU/mL)	C-peptide (ng/mL)	HbA1c	Symptoms of Hypogycemia	Did Patient Take Diazoxide?	Dosage Reccomended to the Patient	Dosage Taken by the Patient
2005	41	29.8	78	2.5				No	Yes	200 mg	N/A
2014	50	33.8	88.5	3.13				No	Yes	200 mg	200 mg
2016	52	31.3	82	2.91			3.9	Loss of consciousness	Yes	200 mg	100 mg
2017	53	31.3	82	2.68	5.66	1.49	4.0	Loss of consciousness	Yes	200 mg	100 mg
2020	56	32.4	85	2.35	5.3	1.34	4.1	No	No	200 mg	
2021	57	32.4	85	1.88	14.2		4.3	No	No	200 mg	

**Table 3:** Current biochemical status of affected patients.

	I.1	II.1	II.3	II.5
SGOT (U/I) normal range (10–50),	23	34	19	NA
SGPT (U/I) (10–50)	17	30	18	NA
GTP (U/I] normal range (8–61)	42	48	11	NA
Total cholesterol (mmol/L) (3.2–5.2)	6.1	4.8	3.2	NA
LDL (mmol/L) (<3.4)	3.6	3.2	1.3	NA
HDL (mmol/L) (>1.0)	2.1	1.3	1.6	NA
TG (mmol/L) (<2.26)	1.1	1.8	0.7	NA
Creatinin (umol/L) (62.0–106.0]	66.3	85.9	75	NA
TSH (uIU/mL) normal range (0.270–4.200)	0.9	1.5	0.6	NA
fT4 (pmol/L) normal range (12.0–22.0)	12.3	NA	14.6	NA

SGOT: aspartate aminotransaminase; SGPT: alanine aminotransaminase; GGT: gamma-glutamyltransferase; LDL: low-density lipoprotein; HDL: high-density lipoprotein; TG: triglycerides; TSH: thyroid-stimulating hormone; fT4: free thyroxine; NA: data not available.

**Table 4:** Summary of metabolic profile results, concomitant symptoms of hypoglycemia and treatment in patient IIA.

Year	Age	BMI	Weight (kg)	Fasting Blood Glucose (mmol/L)	Insulin (uU/mL)	C-peptide (ng/mL)	HbA1c	Symptoms of Hypogycemia	Did Patient Take Diazoxide?	Dosage Reccomended to the Patient	Dosage Taken by the Patient
2014	22	23.1	74	2.54				No	Yes	200 mg	200 mg
2016	24	25.9	83	2.87	15.41			No	No	200 mg	
2020	28	27.2	87	2.39	13.9	2.61	3.8	Occasionally blurred vision	No	200 mg	
2021	29	27.8	89	2.24	27.1		4.2	Fatigue	No	200 mg	

**Table 5:** Clinical characteristics of reported *GCK* cases and affected family members.

	Inheritance Patterns		Type of Mutation	Onset Age	Sex	BW (g)	Pretreatment Plasma Glucose (mmol/L)	Diazoxide Response
		***De novo* Cases**						
1.	*De novo*	[16]	c.191C>A p.Ser64Tyr	1 day	male	4300	2.0	Yes
2.	*De novo*	[10]	c.296G>A p.Trp99Arg	6 years	male	3200	2.7	Partial
3.	*De novo*	[18]	c.589A>G p.Met197Val	9 years	male	4800	2.2	Partial
4.	*De novo*	[10]	c.591G>T p.Met197Ile	1 h	male	4900	2.2	Yes
5.	*De novo*	[13]	c.641A>G p.Tyr214Cys	1 day	female	4400	2	No
6.	*De novo*	[24]	c.1354G>C p.Val452Leu	1 day	male	5900	2.5	Yes
7.	*De novo*	[10]	c.1361-1364 ins CGG p.ins454Ala	1 h	male	4800	2.2	No
		**Familial Cases**						
8.	AD	Patient’s mother: hypoglycemia (2.2 mmol/L), successfully treated with diazoxide [3]	c.194C>T p.Thr65Ile	neonate	male	3100	2.2	Yes
9.	AD	Patient’s mother: NA [4]	c.212T>C, p.Val71Ala	neonate	NA	NA	NA	NA
10.	AD	8 affected family members with variable phenotype. 7/8 managed with diet. Index patient treated with diazoxide and octreotide [25]	c.203G>T p.Gly68Val	months	female	3700	1.6	Yes
11.	AD	The proband’s mother and aunt were asymptomatic mutation carriers [18]	c.269A>G p.Lys90Arg	20 years	female	4600	2.5	NA
12.	AD	The patient’s father had a similar disease course to the index patient [5,6]	c.271C>G p.Val91Leu	1 day	female	macrosomia	1.7	Yes
13.	AD	Patient’s father: asymptomatic hypoglycemia (2.7–3.0 mmol/L), no treatment [3]	c.295T>A p.Trp99Arg	1 day	male	3100	2.4	Partial
14.	AD	The mutation was found in the patient’s sister, father, two paternal uncles, and paternal grandmother, with all but the latter exhibiting fasting hypoglycemia [7]	c.308C>G p.Tyr103Ser	15 years	female	3200	2.8	Yes
15.	AD	The patient’s sister had the same mutation (random glucose level 56 mg/dL, HbA1c 3.9%). Patient’s mother refused genetic screening but also presented with hypoglycemia. [8].	c.538A>G p.Asn180Asp	childhood	female	NA	2.1	Partial
16.	AD	Incidentally discovered, asymptomatic hypoglycemia was observed in the patient’s son (<60 mg/dL) and grandson (46 mg/dL), with the same mutation confirmed [10,26]	c.590T>C p.Met197Thr	44 years	female	NA	2.6	NA
17.	AD	Patient’s father: mutation carrier [7]	c.1165C>G p.Val389Leu	2 years	male	5400	2.9	Partial
18.	AD	Affected brother and mother presented with symptomatic hypoglycemia [9]	c.1324G>A p.Glu442Lys	1 day	female	2800	1.5	Yes
19.	AD	The patient’s father, sister and two children affected with the same mutation, all treated with diazoxide [10,11]	c.1363C>A p.Val455Met	31 years	male	4300	2.5	Yes
20.	AD	Patient’s mother: asymptomatic mutation carrier [12]	c.1367C>T p.Ala456Val	1 day	male	3800	3.1	Yes
		**Unknown family history**						
21.	NA	[6]	c.297G>T p.Trp99Cys	25 years	female	2400	2.9	Yes
22.	NA	Present study	c.295T>C p.Trp99Arg	20 years	male	3800	NA	Partial

AD: autosomal dominant; NA: data not available; BW: Birth weight.

## Data Availability

Not acceptable.
